# A Prospective Multi‐Center Implementation Study to Improve the Diagnosis and Treatment of Benign Paroxysmal Positional Vertigo

**DOI:** 10.1111/acem.70177

**Published:** 2025-11-03

**Authors:** Robert Ohle, Danielle Roy, Elger Baraku, Kashyap Patel, David W. Savage, Sarah McIsaac, Ravinder Singh, Daniel Lelli, Darren Tse, Peter Johns, Krishan Yadav, Jeffrey J. Perry

**Affiliations:** ^1^ The Department of Emergency Medicine Health Sciences North, Northern Ontario School of Medicine, Health Sciences North Research Institute Sudbury Ontario Canada; ^2^ Faculty of Medicine, School of Epidemiology and Public Health University of Ottawa Ottawa Ontario Canada; ^3^ The Faculty of Medicine University of Ottawa Ottawa Ontario Canada; ^4^ The Department of Emergency Medicine Northern Ontario School of Medicine Thunder Bay Ontario Canada; ^5^ Department of Critical Care, Department of Anesthesia Northern Ontario School of Medicine Sudbury Ontario Canada; ^6^ The Department of Neurology Health Sciences North, Northern Ontario School of Medicine, Health Sciences North Research Institute Sudbury Ontario Canada; ^7^ Department of Emergency Medicine University of Ottawa and Ottawa Hospital Research Institute Ottawa Ontario Canada

## Abstract

**Background:**

Benign paroxysmal positional vertigo (BPPV) is the most common cause of vertigo, yet it remains underdiagnosed and undertreated in emergency departments (EDs). Despite evidence‐based guidelines recommending bedside diagnostic maneuvers (Dix‐Hallpike and supine roll test) and canalith repositioning maneuvers (CRMs), these are infrequently utilized, leading to unnecessary imaging, prolonged symptoms, and increased healthcare utilization.

**Objective:**

This study aimed to implement an educational strategy to improve the diagnosis and treatment of BPPV in the ED by increasing adherence to guideline‐based practices.

**Methods:**

We conducted a multicenter interrupted time series study from August 2020 to September 2023. The intervention, developed using the CAN‐Implement framework, included online training, quick‐reference tools, and a mobile app. Due to the COVID‐19 pandemic, in‐person training was canceled. The primary clinical outcome was the proportion of patients receiving the appropriate CRM based on positional test results. Implementation outcomes included fidelity, appropriateness, adoption, penetration, and system impact, reported using the Standards for Reporting Implementation Studies (StaRI) guidelines.

**Results:**

We included 1682 patients (1252 pre‐intervention, 430 post‐intervention). There was no significant change in the primary outcome (appropriate CRM use, OR = 1.08, 95% CI: 0.76–1.40). However, selective CT use improved (OR = 1.29, 95% CI: 1.09–1.49), supine roll testing increased from 14.2% to 23.5%, and neurology consults decreased from 7.1% to 4.0%. Documentation of diagnostic test descriptors improved, while neurological exam documentation declined.

**Conclusion:**

The intervention did not significantly increase appropriate CRM use but led to improvements in selective imaging, neurology consultation, and horizontal canal testing. Provision of educational tools alone was insufficient to overcome identified environmental barriers. To effectively improve BPPV management in the ED, future efforts should combine hands‐on training with system‐level supports and workflow integration.

## 1 Introduction

Dizziness/vertigo is a common presenting complaint to the emergency department (ED) [1, 2, 3]. The vast majority of cases are caused by one of several self‐limiting processes [4]. The most common is benign paroxysmal positional vertigo (BPPV) [4]. This condition is characterized by brief episodes of dizziness/vertigo/imbalance brought on by head movement. It is caused by the displacement of small‐calcified stones, or otoconia, in the patient's inner ear. The description as “benign” is a misnomer, as this condition can result in significant societal (e.g., lost days at work) and personal impacts (inability to conduct activities of daily living/drive, increased falls risk) [5].

In BPPV, there are three subtypes: anterior canal, posterior canal, and horizontal canal. The type depends on the semicircular canal into which the otoconia are displaced. Posterior canal BPPV is the most common; this is diagnosed with the Dix‐Hallpike maneuver and is treated with the Epley maneuver. The second most common is horizontal canal BPPV; this is diagnosed with the supine roll test and treated with the Gufoni maneuver. Therefore, BPPV can be accurately diagnosed at the bedside with simple bedside tests (Dix‐Hallpike or supine roll test), negating the need for advanced diagnostic imaging. After diagnosis, the condition can also be effectively treated at the bedside through canalith repositioning maneuvers (CRMs), such as the Epley or Gufoni maneuvers [6].

Currently, the vast majority of patients who are diagnosed with BPPV are given the diagnosis based on symptoms alone. They do not have any bedside diagnostic tests performed in the ED, nor do they undergo CRMs to reduce prolonged symptoms [7]. Neuroimaging is not indicated in patients with BPPV; however, one in three of those diagnosed will undergo computed tomography of the head, leading to unnecessary radiation exposure and prolonged ED visits [8].

**FIGURE 1:**
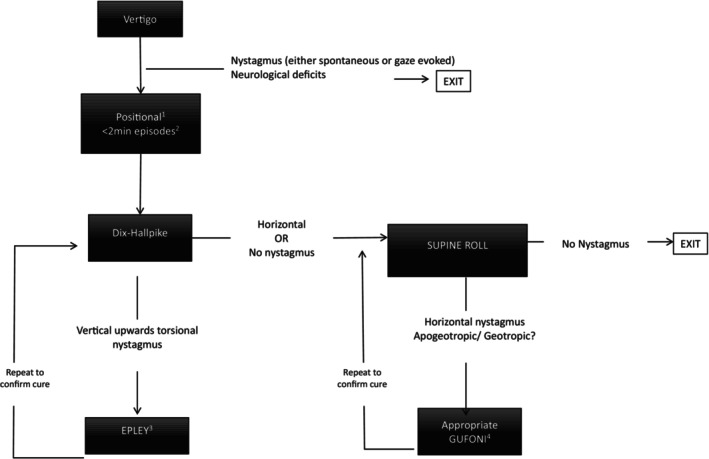
BPPV best practice algorithm. ^1^Positional—commonly episodes provoked by head movements such as rolling over/getting out/in bed, bending over or tilting head backwards. ^2^< 2 min—episodes are brief and severe, may still feel somewhat off in between the episodes. ^3^Repeat Dix‐Hallpike after Epley if still positive repeat EPLEY. ^4^The appropriate Gufoni maneuver is based on whether the nystagmus is beating away (apogeotropic) or towards (geotropic) the ground and which ear is affected. After the maneuver repeat the supine roll test. If no nystagmus is present exit the algorithm.

There is evidence‐based clinical practice guidelines that provide a diagnostic pathway for the diagnosis and treatment of BPPV. A national guideline recommending the Dix‐Hallpike or supine roll test, and CRMs was published in 2008 and updated in 2017 by the American Academy of Otolaryngology‐Head and Neck Surgery and the American Academy of Neurology [6, 9]. The diagnostic and therapeutic pathway for BPPV in the 2017 guidelines (Figure 1) is based on multiple systematic reviews supporting the Dix‐Hallpike or supine roll tests, and CRMs. The guideline is supported by numerous randomized controlled trials demonstrating CRMs can improve and often resolve BPPV symptoms. In these studies, 61 to 80% of treated patients had resolution after just one treatment compared with 10%–48% of untreated patients. These effect sizes translate into an impressive number needed to treat of 1.4 [6, 9].

However, guidelines and research are only useful if physicians successfully implement them into practice. Previous studies have found that both diagnostic and therapeutic maneuvers are often performed on the wrong patients. A purposefully designed implementation strategy may help improve the consistent use of best practice guidelines.

Our objective was to design and implement an educational strategy using a guideline‐based algorithm to improve the diagnosis and treatment of BPPV. We hypothesized that our educational strategy would increase the utilization of treatment maneuvers for patients diagnosed with BPPV.

## 2 Methods

**FIGURE 2:**
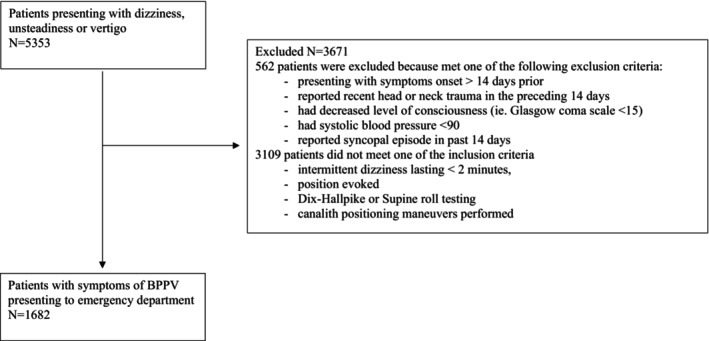
Flow diagram of the study cohort.

Multicenter interrupted time series at three academic emergency departments in Ontario, Canada (Health Sciences North, Sudbury (Unit A) and The Ottawa Hospital (Unit B—Civic and General Campus), Ottawa) between August 1st 2020 and December 15th 2022. We included patients presenting to the emergency department with symptoms of BPPV (Table 1; Figure 2).

**TABLE 1:** Pre‐ and post‐intervention patient characteristics.

Characteristics	Pre‐intervention (*n* = 1252)	Post‐intervention (*n* = 430)
Age, years	57.8 ± 17.2	57.3 ± 17.3
Sex, male	506 (40.4)	172 (40.0)
Diastolic blood pressure, mmHg	82.2 ± 11.2	80.9 ± 11.8
Systolic blood pressure, mmHg	140.6 ± 23.6	141.7 ± 22.7
BPPV diagnosis	546 (43.6)	205 (47.7)
Presenting complaint		
Vertigo	980 (78.3)	321 (74.6)
Dizziness	895 (71.5)	309 (71.9)
Lightheaded	274 (21.9)	95 (22.1)
Unsteadiness	359 (28.7)	170 (39.5)
History		
Gradual	150 (12.0)	54 (12.6)
Abrupt	1023 (81.7)	349 (81.2)
Movement triggered	942 (75.3)	279 (65.2)
Dizziness lasting less than 2 min	563 (45.2)	209 (52.4)
Neurological deficits	131 (10.7)	55 (13.0)
Past medical history		
Hypertension	435 (34.9)	134 (31.2)
Hypercholesterolemia	190 (15.3)	27 (6.3)
Diabetes	177 (14.2)	46 (10.7)
Atrial fibrillation	56 (4.5)	18 (4.2)
Previous stroke	44 (3.5)	13 (3.0)
Previous TIA	32 (2.6)	10 (2.3)
Ischemic heart disease	69 (5.6)	16 (3.7)
Connective tissue disease	57 (4.6)	4 (0.9)
Ototoxic drugs	113 (9.1)	4 (0.9)
Wernicke's	0	0
Herpes zoster	0	1 (0.2)

*Note:* Continuous variables expressed as mean ± SD and categorical variables expressed as *n* (%).

### 2.1 Inclusion Criteria

- Age ≥ 18 years
- Clinical suspicion for BPPV, defined as any one of the following:
  ○ Intermittent dizziness lasting < 2 min
  ○ Position evoked
  ○ Dix‐Hallpike or supine roll, or CRMs performed

### 2.2 Exclusion Criteria

- Dizziness > 14 days
- Glasgow coma scale < 15
- Cervical spine pathology that limits neck manipulation
- Systolic blood pressure < 90.

The study had five phases.

#### 2.2.1 Barriers and Facilitators' Assessment

To evaluate the barriers and facilitators impacting the implementation of the BPPV algorithm, we assessed the local emergency department culture, organizational structure, and feedback on the algorithm's usability. This was achieved through semi‐structured interviews with emergency room physicians across three sites. Purposive sampling was employed to ensure diverse perspectives, and data collection continued until thematic saturation was reached.

The interview questions explored current practice patterns, familiarity with the BPPV algorithm, perceptions regarding its utility, and perceived physician‐ and system‐level barriers and facilitators. The responses were analyzed using the *Theoretical Domains Framework* (TDF) to systematically categorize findings into 14 domains. The framework facilitated a structured content analysis, which was further used to inductively generate overarching themes.

The analysis followed a three‐step approach:

1. *Initial coding*: Interview transcripts were deductively coded into TDF domains. Two researchers independently coded the first three interviews to ensure consistency and alignment with the framework.
2. *Belief statement development*: Coded excerpts were grouped into belief statements that captured recurring ideas, such as “time constraints limit guideline adherence” and “training materials improve confidence in maneuvers.”
3. *Theme identification*: Belief statements were synthesized into sub‐themes, which were further organized into four overarching themes. This step allowed the integration of novel insights that emerged during analysis.

#### 2.2.2 Development of a Targeted Education Strategy

A targeted education strategy was developed based on the pre‐implementation phase barrier and facilitator assessments and informed by the CAN‐Implement process [10]. A Blended Learning Design with five Key Elements guided the development of the education strategy [11]. The education strategy included the following elements: In‐person training (1–2 h session); online training (online access to the algorithm with embedded links to brief [< 20 s] informative videos); assessment of knowledge (post‐training self‐assessment survey); and reference material (hard copy and online reference material related to the training and to support the use of the BPPV algorithm).

#### 2.2.3 Baseline Data Collection

Baseline data was collected via a retrospective chart review at each participating site. Patients were identified through a presenting complaint of dizziness or vertigo or a discharge diagnosis related to vertigo (i.e., vertigo, unsteadiness, BPPV). These charts were then screened for inclusion/exclusion criteria. Two trained reviewers used a standardized data collection sheet to extract data from charts based on methods described by Jansen et al. [12]. When a variable was not documented, it was coded as not done. Diagnosis of BPPV was based on ED physician impression defined by discharge diagnosis.

#### 2.2.4 Implementation of Education Strategy

We planned on both in‐person and digital educational components. Specifically, we planned to conduct 1–2 h in‐person training sessions with physicians and emergency medicine residents to provide hands‐on practice with diagnostic and therapeutic maneuvers for BPPV. These sessions were to be supported by Supporting Information, including printed reference tools and a mobile app.

All physicians and emergency medicine residents practicing in the emergency department were invited to participate; participation was voluntary. Training consisted of an online training video, a pre‐ and post‐survey to identify knowledge gaps, and then empower participants by demonstrating a successful increase in knowledge. Posters were deployed, including a link to an online app that guided the user through the algorithm, providing short informational videos demonstrating diagnostic and therapeutic maneuvers from BPPV. The educational intervention was rolled out and completed over a 2‐week period.

#### 2.2.5 Post‐Implementation Data Collection

Once the training was completed and physicians were familiar with the training resources, the post‐implementation data collection assessed the primary and secondary outcomes. Documentation related to adherence to the BPPV algorithm was collected prospectively post‐implementation. Physicians were asked to screen patients presenting with dizziness, vertigo, or unsteadiness and, when patients met eligibility, complete a prospective data collection sheet. When a sheet was not filled, the chart was reviewed retrospectively. When a variable was not documented, it was coded as not done or not present. Diagnosis of BPPV was based on ED physician impression defined by discharge diagnosis.

### 2.3 Primary Clinical Outcome

The primary outcome was the proportion of patients receiving the appropriate CRM based on the result of a Dix‐Hallpike or supine roll test. Specifically:

- If both tests were negative, no maneuver should be performed.
- If the Dix‐Hallpike test was positive, the Epley maneuver should be performed.
- If the supine roll test was positive, the Gufoni maneuver should be performed.

### 2.4 Implementation Outcomes

Implementation outcomes were structured using domains from Proctor's implementation outcomes framework and are reported in accordance with the Standards for Reporting Implementation Studies (StaRI) guidelines [13, 14].

1. Fidelity (algorithm adherence)
  ○ *Neurological deficit documentation*: Proportion of BPPV diagnoses with documented absence of neurological deficits, and among non‐BPPV cases, proportion with documentation of a neurological exam (either with or without findings).
  ○ *Appropriate test use*: Proportion of patients with BPPV‐consistent symptoms (e.g., positional vertigo < 2 min) who underwent a Dix‐Hallpike and/or supine roll test.
  ○ *Test descriptor documentation*: Proportion of patients with documented nystagmus characteristics (e.g., torsional, horizontal), worsening dizziness, or no change during positional testing.
2. Appropriateness
  ○ *Selective CT imaging*: Proportion of patients receiving a CT head scan in whom imaging was likely indicated (defined as negative positional tests, symptoms lasting > 2 min, or final diagnosis inconsistent with BPPV).
3. Adoption
  ○ *Use of canalith repositioning maneuvers*: Proportion of eligible patient encounters in which a CRM (Epley or Gufoni) was performed.
4. Penetration
  ○ *System reach*: Proportion of patients presenting with BPPV‐consistent symptoms who received any diagnostic positional test, regardless of test result or subsequent treatment.
5. System Impact
  ○ *Resource utilization*: Number and proportion of patients receiving advanced imaging (CT, CTA, MRI), referrals to specialty services (neurology, ENT, stroke prevention), or hospital admission for dizziness.

### 2.5 Data Analysis

Descriptive statistics were summarized using mean and standard deviation (SD) or median and interquartile range (IQR) for continuous data and counts and percentages for categorical data. We used chi‐squared and *t*‐tests to assess differences in patient characteristics pre‐ and post‐intervention.

To assess trends in compliance with primary and secondary outcomes over the 29‐month period, we logit‐transformed monthly proportions into log‐odds and used segmented linear regression. To accommodate hospital heterogeneity, we fit separate segmented models for each unit and pooled estimates by inverse variance as described by Gebski et al. [15]. We also pooled across hospitals using a “stacked” model [15]. We hypothesized no burn‐in period for the effect of the intervention since, following the intervention, healthcare providers may have immediately applied communicated content. Segmented regression included coefficients for pre‐intervention slope, immediate level change, and change in slope post‐intervention, adjusted for mean age. We assessed models for autocorrelation and seasonality using the Durbin–Watson test (lag 12) and Dickey–Fuller unit root test. All segmented models were fit using maximum likelihood estimation, corrected for first‐order autocorrelation. Goodness of fit was examined by comparing observed versus predicted proportions. All analyses were conducted in SAS Version 9.4.

### 2.6 Sample Size

We anticipated an average of 25 patients per month at the smallest site (annual 75,000 ED visits). Retrospective data collection covered 43 months (1075 patients) and post‐intervention 9 months (225 patients). Minimum recommendations for robust interrupted time series are 8–12 pre and 8–12 post measurements. Power calculations require estimates for mean square error, serial autocorrelation, intercept, and slope. As we had no advance estimates, we assumed intercept 0.07, no slope, and no autocorrelation. Based on these assumptions, we had 80% power to detect an absolute increase from 0.07 pre to 0.12 post using a two‐tailed test at the 0.05 level of significance. This study was approved by Clinical Trials Ontario (Health Sciences North Research Ethics Board).

The manuscript was prepared using the StaRI checklist (Appendix S1).

## 3 Results

### 3.1 Study Population

We included 1252 patients in the pre‐intervention period and 430 in the post‐intervention period. The pre‐intervention period spanned from August 1, 2020, to November 30, 2022. The intervention was implemented over a two‐week period in December 2022, and the post‐intervention data collection occurred from January 1 to September 30, 2023. There were no significant differences in age, sex, or diagnosis of BPPV between the two cohorts.

### 3.2 Barriers and Facilitators' Assessment

Through semi‐structured interviews with 11 emergency department physicians, we identified four overarching themes influencing the uptake of the BPPV guideline. First, there was considerable variability in awareness and use of the algorithm. Many physicians were unfamiliar with the tool, though they acknowledged its potential utility, with one stating, “I've heard of it, but it's not something we routinely use because we often default to imaging.” Second, most participants recognized the clinical benefits and expressed motivation to use the algorithm, highlighting its potential to streamline care and improve diagnostic accuracy. One physician noted, “The Dix‐Hallpike and Epley maneuvers are incredibly effective when applied correctly.” Third, physicians emphasized patient comfort and environmental constraints as key barriers, such as symptom severity, nausea, or space limitations in a crowded ED. Finally, the need for accessible educational materials was repeatedly mentioned; clinicians preferred quick‐reference tools like videos or mobile apps to build confidence, especially in managing horizontal canal BPPV, which is encountered less frequently.

### 3.3 Fidelity and Adaptations

Due to the COVID‐19 pandemic, the implementation of the education was forced to be modified by canceling the in‐person training. The majority of physicians (82.5%, 99/120) engaged with the educational intervention, which included an online module, posters, and an app. There was a significant improvement in knowledge scores post‐intervention, increasing from 72.7% to 84%, primarily due to a better understanding of horizontal canal BPPV and supine roll testing (accuracy increased from 62% to 87.5%). Due to the COVID‐19 pandemic, in‐person training sessions were canceled and replaced with digital content. Other than this adaptation, fidelity to the planned rollout was maintained through consistent deployment of the other educational materials across all sites (Tables 2 and 3).

**TABLE 2:** Random effects model odds ratios with 95% confidence intervals.

Outcome	Pre‐intervention trends	Post‐intervention trends
Performed appropriate canalith repositioning maneuvers (Gufoni/Epley)	0.95 (95% CI: 0.79, 1.11)	1.08 (95% CI: 0.76, 1.40)
Neurological deficit documentation	1.29 (95% CI: 1.07, 1.51)	0.84 (95% CI: 0.56, 1.12)
Appropriate diagnostic test administration	1.03 (95% CI: 0.85, 1.21)	0.96 (95% CI: 0.41, 1.51)
Reported the appropriate test descriptors	0.90 (95% CI: 0.76, 1.04)	1.21 (95% CI: 0.99, 1.43)
Selective CT imaging	0.93 (95% CI: 0.79, 1.07)	1.29 (95% CI: 1.09, 1.49)

**TABLE 3:** Comparison of diagnostic, therapeutic tests and resource utilization.

	Pre‐intervention (*n*, %)	Post intervention (*n*, %)
Diagnostic tests	996 (79.5%)	330 (77.4%)
Dix‐Hallpike test administered	984 (78.6)	328 (76.3)
Supine roll test administered	178 (14.2)	101 (23.5)
Therapeutic tests	374 (29.9)	135 (31.4)
Epley maneuver administered	358 (28.6)	127 (29.5)
Gufoni maneuver administered	23 (1.8)	9 (2.1)
Resource utilization		
CT performed	335 (26.8)	135 (31.4)
MRI performed	25 (2.0)	13 (3.0)
CTA performed	182 (14.5)	86 (20.0)
Neurology	89 (7.1)	17 (4.0)
ENT	16 (1.3)	5 (1.7)
Stroke prevention clinic	24 (1.9)	9 (2.1)
Admitted	49 (3.9)	20 (4.6)

### 3.4 Primary Clinical Outcome

The proportion of patients receiving an appropriate canalith repositioning maneuver based on diagnostic test result increased post‐intervention, but this change was not statistically significant (OR = 1.08, 95% CI: 0.76–1.40). This outcome reflects patient‐level alignment with guideline‐recommended care and was selected as the key clinical effectiveness measure.

### 3.5 Implementation Outcomes

#### 3.5.1 Uptake

The use of CRMs (Epley or Gufoni) increased modestly from 29.9% to 31.4% post‐intervention, though this change was not statistically significant (OR = 1.08, 95% CI: 0.76–1.40).

#### 3.5.2 Adoption

Documentation of test descriptors (e.g., nystagmus type) improved, with odds increasing post‐intervention (OR = 1.21, 95% CI: 0.99–1.43). However, documentation of neurological assessments declined (OR = 0.84, 95% CI: 0.56–1.12), and use of Dix‐Hallpike or supine roll tests remained stable (OR = 0.96, 95% CI: 0.41–1.51).

#### 3.5.3 Penetration

The proportion of patients undergoing positional testing did not significantly change overall, though supine roll testing increased from 14.2% to 23.5%. Dix‐Hallpike test usage remained unchanged.

#### 3.5.4 Appropriateness

Selective CT use improved following the intervention (OR = 1.29, 95% CI: 1.09–1.49), suggesting better alignment with clinical guidelines. However, there was a rise in CT angiography (CTA) use from 14.5% to 20.0%, and overall CT rates remained unchanged.

#### 3.5.5 System Impact

Neurology consultation rates decreased from 7.1% to 4.0%. No significant changes were observed in MRI or overall CT usage, referrals to ENT or stroke prevention clinics, or admission rates.

#### 3.5.6 Unintended Consequences

**FIGURE A1:**
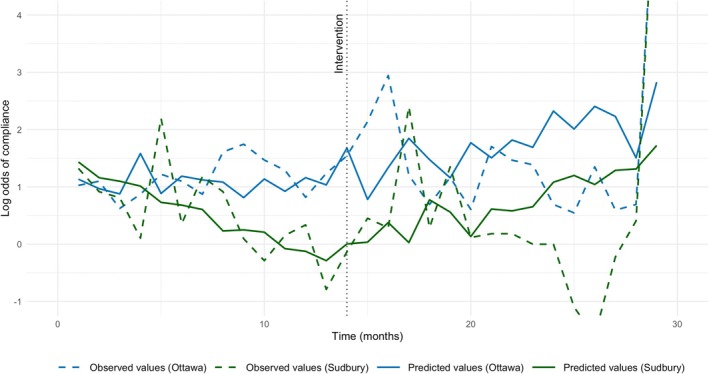
Trend in appropriate canalith positioning maneuver over time at Unit A (Health Sciences North Sudbury—green) and Unit B (The Ottawa Hospital [Civic and General Campus] blue). The dashed line represents observed values, and the solid line represents predicted.

The improvements in documentation and selective imaging may reflect enhanced awareness and record‐keeping rather than changes in bedside behavior. The increase in CTA use, despite stable CT rates overall, may reflect institutional or temporal trends unrelated to the intervention. Although confidence in managing horizontal canal BPPV improved, Gufoni maneuver use did not increase, possibly due to persistent workflow and perceived space barriers. No adverse events were reported. The intervention did not increase inappropriate imaging or admissions, but its potential to reduce resource use remains unproven in this context (Table A1; Figure A1).

## 4 Discussion

In this multicenter implementation study, we found that an educational intervention designed to increase appropriate use of canalith repositioning maneuvers for benign paroxysmal positional vertigo did not significantly improve our primary clinical outcome—performance of a CRM when indicated based on positional testing. Despite enhanced clinician knowledge and increased documentation of key test descriptors, adoption of appropriate therapeutic maneuvers remained unchanged. Table A2 demonstrates that physicians were good at not performing the therapeutic maneuver when it was not indicated (90.2%). However, when a CRM was indicated, they were only performing on less than half of patients (43.9%), and this did not change in the post‐intervention period.

However, implementation outcome analysis noted improvements across several implementation outcomes, including fidelity (e.g., improved documentation of nystagmus type and diagnostic test descriptors), appropriateness of selective CT utilization, and modest gains in penetration (e.g., increased use of horizontal canal testing such as the supine roll). A small reduction in neurology referrals was also noted, suggesting possible system‐level impact. These implementation outcome findings indicate that while the intervention addressed certain knowledge gaps and documentation behaviors, it did not overcome key contextual and operational barriers within the emergency department environment. This aligns with broader implementation science literature demonstrating that education alone is rarely sufficient to produce meaningful and sustained clinical behavior change, especially in high‐functioning or resource‐constrained environments [16].

When examined through the lens of implementation science, several contextual and process‐related barriers likely contributed to the intervention's limited impact on our primary clinical outcome. According to the Consolidated Framework for Implementation Research (CFIR), both inner setting constraints and process limitations played critical roles [17]. Space and time limitations in the emergency department were repeatedly cited in interviews as major challenges, reflecting barriers within the “available resources” and “compatibility with workflow” constructs. These were not addressed as part of our intervention. The cancellation of in‐person, hands‐on training—due to the COVID‐19 pandemic—likely weakened the “intervention process,” particularly around skill acquisition and engagement, which are essential for translating knowledge into action. Acceptability may have also influenced implementation success. Although clinicians received the same educational materials, the supine roll test and Gufoni maneuver were adopted at different rates across sites. This variation suggests that individual clinician preferences, confidence, or beliefs about the maneuver's utility likely shaped uptake. In particular, the Gufoni maneuver—used for horizontal canal BPPV—is less familiar and more technically demanding than the Epley maneuver, which may have reduced its perceived feasibility or comfort level among some providers. Additionally, local norms or differing perceptions of value for horizontal canal testing may have affected clinicians' willingness to apply these techniques.

While our preparatory work identified several barriers to optimal BPPV management—such as limited awareness, discomfort with maneuver execution, and documentation gaps—these were largely knowledge‐ and confidence‐based and therefore pointed to an educational intervention as a logical and feasible first step. At the time, the intervention aligned with available resources and was selected to be scalable across three sites. However, we now recognize that the barriers uncovered may have warranted more tailored, site‐specific strategies to address distinct inner setting contexts, such as differences in workflow, staffing, or local norms. The divergent implementation outcome patterns observed across sites suggest that a one‐size‐fits‐all approach may have been suboptimal. Future interventions should consider site‐level readiness, variation in baseline performance, and differential acceptability or feasibility of specific maneuvers when designing implementation strategies. Moreover, a hybrid effectiveness‐implementation design with iterative adaptation may have better captured and responded to these dynamic local factors. These findings underscore that educational interventions, when decoupled from operational redesign and reinforcement mechanisms, may be insufficient to shift practice—especially in high‐acuity, high‐throughput clinical environments. Future strategies should more explicitly address these contextual factors through integrated decision support, protected clinical time, and embedded champions to support sustained adoption.

### 4.1 Previous Studies

There are few studies assessing the appropriateness of diagnostic or therapeutic maneuvers for BPPV. Kerber et al. conducted a step wedge RCT implementing an educational intervention to improve the treatment of patients with BPPV [18]. The overall use of diagnostic or therapeutic maneuvers in the pre‐intervention period was 7%, and the post‐intervention rate was 17%. They did not assess if the test was performed on the appropriate patient. A 2022 study by Del Risco et al. reported that 41.5% of patients underwent a Dix‐Hallpike, only 4.1% underwent a supine roll test, 11.1% underwent an Epley maneuver, and none underwent maneuvers for a horizontal canal BPPV [19]. A retrospective chart review by Neely et al. found that only 45% of patients diagnosed with BPPV had a diagnostic maneuver, and of these, only 41% had a therapeutic maneuver. There was no record of a supine roll test being performed [18, 19]. They do not report on the presenting symptoms of the patients or comment on the appropriateness of the diagnostic tests being performed. We had a higher rate of diagnostic tests being performed (78%) and a comparable number of therapeutic maneuvers (30%) at baseline. Neither significantly changed post intervention. This may reflect the limited effectiveness of an online educational intervention in further improving rates of maneuver usage in a high‐use setting. Gerlier et al. implemented a two‐tiered educational intervention, combining hands‐on training and an online decision support tool, following the 2023 GRACE‐3 emergency medicine clinical practice guideline release [20]. In their study, CRMs—primarily Epley or a different horizontal canal CRM maneuver (Lempert‐barbecue) following a positive positional test—were performed in 90.2% of diagnosed BPPV cases, compared to 57.7% pre‐intervention [20]. In our study, when a CRM was indicated based on test results, it was performed in only 43.9% of cases, reflecting more modest uptake. This difference may relate to the in‐person, hands‐on format of their training, which is likely critical for skill acquisition, whereas our intervention was delivered virtually due to pandemic restrictions, likely limiting skill transfer.

In our interviews with clinicians, we found that the majority felt comfortable with the use of the Dix‐Hallpike (82%) and Epley maneuver (73%), but only 1/3 were comfortable with the supine roll, and none were comfortable with the Gufoni maneuver. We found that the most significant impact of our intervention was in the use of the supine roll test to diagnose horizontal canal BPPV. There was no increase in the use of the Gufoni maneuver. There are four subtypes of horizontal canal BPPV. You may have nystagmus that beats towards the ground (geotropic) or away from the ground (apogeotropic), and it can either affect the left ear or right ear. There is a particular variation of the Gufoni maneuver to be used depending on the ear affected and if it is apogeotropic or geotropic. Although our intervention improved the comfort in using the supine roll, it did not translate into increased usage of the therapeutic Gufoni maneuver. The Kerber study did not report on the change in the usage of a particular CRM, and neither the Neely study nor the Del Resco study report on the usage of the Gufoni maneuver [18, 19]. While the Grelier study noted 100% of supine roll test positive patients underwent Lempert‐barbecue pre‐ and post‐intervention, they noted a marked increase in the use of the supine roll test (4/166 pre vs. 78/218 post), leading to a more modest increase in the proportion of the Lempert‐barbecue maneuvers performed for horizontal canal BPPV (2/166 pre vs. 13/216 post). Historically, it was felt that horizontal canal BPPV was rare; this may account for the fact that the supine roll or Gufoni maneuver is rarely mentioned in medical education. However, more recent studies identify that the rate of horizontal canal BPPV could be as high as 9%–46% [8, 21, 22, 23]. The variation in these rates may be attributed to the fact that in these studies, providers were educated about horizontal canal BPPV and trained on how to assess for the diagnosis. This indicates that the test and treatment for horizontal canal BPPV is likely underperformed and underreported.

The intervention did not impact the overall use of CT pre‐ and post‐intervention; however, there was an observed increase in the proportion of CTs performed as CTA versus plain CT. This finding is unadjusted for temporal trends and may reflect a general trend over time towards increased CTA utilization. The Kerber study showed a 37% post‐intervention CT usage, higher than our 31% post‐intervention rates, which were unchanged from pre‐intervention. This likely reflects the different image utilization rates between Canada and the United States, with the latter, on average, reporting higher utilization of computer tomography in the emergency department. For comparison, 36% underwent neuroimaging in the Australian Neely study, and 25% underwent neuroimaging post‐intervention in the French Grelier study. This remains an area for targeted intervention in order to improve resource utilization.

Importantly, the intervention demonstrated an overall improvement in the odds of adherence to appropriately selective CT imaging. CT imaging was deemed appropriate when performed only in patients without a suspicion of BPPV, defined as those with negative Dix‐Hallpike and/or supine roll tests, symptoms lasting > 2 min, or discharge diagnoses unrelated to BPPV. Interestingly, Unit B exhibited the strongest impact, with a 42% increase in selective CT imaging adherence, reflecting meaningful improvements in imaging practices following the intervention. In contrast, Unit A showed a more modest 17% increase, which was not significant, suggesting variability in the intervention's effectiveness across units. This variability reflects differences in site‐level implementation success, a common challenge in real‐world studies. Differences in workflow compatibility, clinician engagement, and baseline practices likely contributed to the differential impact across units. While the aggregate results highlight an overall effect of the intervention, the differences between units suggest that targeted strategies may be necessary to optimize outcomes in specific contexts to reduce unnecessary CT scans in patients with clear clinical indicators of BPPV.

Our admission rate was 4.6%. This is lower than a 2022 study exploring factors associated with the admission of patients for BPPV [24]. In the Kerber study, 15% of patients were admitted. We found no change in admission post‐intervention, though Grelier noted a decrease from 17.5% to 6.9%. Not finding a change from an already low admission rate in our cohort is not surprising, as admission for patients with symptoms consistent with BPPV is uncommon.

Referral to neurology decreased post‐intervention (7% to 4%) in our study, and Grelier reported a decrease in specialist consultation (neurology and otolaryngology) from 21% to 15%. Due to the small number of consultations in each time period, we were unable to perform a time series analysis on this outcome. Any univariate comparisons may suffer from potential temporal trends, particularly given the overlap with COVID‐19 and potential implications on consult availability and other metrics related to the study.

### 4.2 Clinical Implications

The educational intervention and online app, both freely available (see Section 2), offer a scalable and resource‐efficient solution for improving adherence to best practices. However, the effectiveness of such interventions will likely depend upon hands‐on training sessions and a comprehensive implementation approach that addresses perceived barriers of ED providers and their willingness to adopt new workflows. Implementing the best practice algorithm has the potential to reduce unnecessary neurology consultations while improving provider comfort with underutilized diagnostic maneuvers for BPPV, such as the supine roll test. Additionally, the intervention demonstrated a potential improvement in appropriate selective CT utilization, emphasizing its role in promoting evidence‐based imaging practices.

It is important to note that while educational interventions can address knowledge gaps and improve practice patterns, their impact may be limited without concurrently addressing environmental and operational constraints faced by ED physicians. These constraints include time pressures, patient volumes, and access to dedicated spaces for performing diagnostic maneuvers. As demonstrated by the variability in outcomes between units, tailored approaches that consider individual emergency departments' unique challenges and workflows may be required to optimize the intervention's effectiveness.

### 4.3 Limitations

The study design was quasi‐experimental, and the rollout of our intervention was not randomized. Our sites had a high baseline usage of diagnostic maneuvers; this may have impacted our ability to detect a change in practice (ceiling effect). When a variable was not documented, it was coded as not done. Therefore, it is possible that providers performed diagnostic and/or therapeutic maneuvers and did not document these. Similarly, it is possible that appropriate descriptors for each test were recognized but just not documented. This could bias us towards finding no impact. Due to the limited sample size, we were not able to adjust for time trends when assessing the increase in the use of the supine roll or the decrease in neurology consultation. We did not collect data on adverse events related to the maneuvers. However, previous studies have shown no change in missed serious diagnoses or adverse events with an intervention to improve the diagnosis and treatment of patients with BPPV [18].

Our inclusion criteria were based on what was documented by providers. Therefore, we may have missed some eligible patients; this would artificially increase the proportion of patients deemed to have received a diagnostic or therapeutic maneuver. Our definition of BPPV would not include those with longer durations of dizziness episodes and therefore would miss some atypical cases of BPPV. Our study did not address one of the identified barriers, namely the emergency department physical environment. With the decrease in usable emergency department space that most centers are experiencing with more boarded patients, providers can find it increasingly challenging to provide appropriate care for patients with BPPV. Finally, one of the outcomes of interest noted in the Grelier study (a mean ED length of stay decrease from 246 to 137 min) was not measured in our study, but may be a compelling reason to pursue this intervention in other EDs.

## 5 Conclusion

An educational intervention in emergency departments with high baseline maneuver utilization did not significantly improve the primary clinical outcome. However, improvements in selective CT use, documentation practices, and diagnostic testing for horizontal canal BPPV demonstrate partial success across key implementation outcomes. Future strategies to fully realize the potential of best practice guidelines for BPPV management should combine hands‐on educational interventions with system‐level redesign, which could include decision support and operational enablement.

## Author Contributions

R.O. designed the study and wrote the first draft of the manuscript. D.R. performed the analysis. E.B. and K.P. performed data collection and cleaning. S.M. provided methodological support for education intervention. R.S., D.L., D.T., P.G. were content experts and provided input on the educational intervention, results interpretation and manuscript editing. J.J.P. and K.Y. provided methodological input and manuscript editing.

## Conflicts of Interest

The authors declare no conflicts of interest.

## Supporting information


**Appendix S1:** acem70177‐sup‐0001‐AppendixS1.docx.

## Data Availability

The data that support the findings of this study are available on request from the corresponding author. The data are not publicly available due to privacy or ethical restrictions.

## Appendix A

**TABLE A1:** Random effects model odds ratios with 95% confidence intervals in Health Sciences North (Unit A) and Ottawa Hospital (Civic and General Campus) Unit B.

Outcome	Unit	Pre‐intervention trends	Post‐intervention trends
Performed appropriate canalith repositioning maneuvers (Gufoni/Epley)	Unit A	1.01 (95% CI: 0.81, 1.21)	1.08 (95% CI: 0.78, 1.38)
	Unit B	0.87 (95% CI: 0.61, 1.13)	1.29 (95% CI: 0.94, 1.64)
	Combined (random effects)	0.95 (95% CI: 0.79, 1.11)	1.08 (95% CI: 0.76, 1.40)
Reported the appropriate test descriptors (e.g., worsening dizziness, no nystagmus)	Unit A	0.98 (95% CI: 0.72, 1.24)	1.13 (95% CI: 0.75, 1.51)
	Unit B	0.86 (95% CI: 0.68, 1.04)	1.25 (95% CI: 0.99, 1.51)
	Combined (random effects)	0.90 (95% CI: 0.76, 1.04)	1.21 (95% CI: 0.99, 1.43)
Reported neurological deficits appropriately	Unit A	1.14 (95% CI: 0.86, 1.42)	0.84 (95% CI: 0.46, 1.22)
	Unit B	1.51 (95% CI: 1.19, 1.83)	0.85 (95% CI: 0.41, 1.29)
	Combined (random effects)	1.29 (95% CI: 1.07, 1.51)	0.84 (95% CI: 0.56, 1.12)
Appropriate use of diagnostic tests (Dix‐Hallpike, Supine Roll)	Unit A	1.14 (95% CI: 0.86, 1.42)	0.72 (95% CI: 0.31, 1.13)
	Unit B	0.95 (95% CI: 0.71, 1.19)	1.24 (95% CI: 0.90, 1.58)
	Combined (random effects)	1.03 (95% CI: 0.85, 1.21)	0.96 (95% CI: 0.41, 1.51)
Selective CT imaging	Unit A	0.97 (95% CI: 0.77, 1.17)	1.17 (95% CI: 1.09, 1.45)
	Unit B	0.88 (95% CI: 0.68, 1.08)	1.42 (95% CI: 1.14, 1.70)
	Combined (random effects)	0.93 (95% CI: 0.79, 1.07)	1.29 (95% CI: 1.09, 1.49)

**TABLE A2:** 2 × 2 Table demonstrating the distribution of appropriate versus inappropriate use of canalith repositioning maneuvers.

	Canalith repositioning maneuvers indicated (*n* = 856)	Canalith repositioning maneuvers not indicated (*n* = 1134)
Canalith repositioning maneuvers performed	376 (43.9%)	108 (9.5%)
Canalith repositioning maneuvers not performed	480 (56.1%)	1026 (90.5%)
